# Relationship Between Obesity, Physical Activity, and Cardiorespiratory Fitness Levels in Children and Adolescents in Bosnia and Herzegovina: An Analysis of Gender Differences

**DOI:** 10.3389/fphys.2018.01734

**Published:** 2018-11-28

**Authors:** Haris Pojskic, Bahareh Eslami

**Affiliations:** ^1^Swedish Winter Sports Research Centre, Mid Sweden University, Östersund, Sweden; ^2^Department of Sports Science, Linnaeus University, Kalmar, Sweden; ^3^Department for Health Sciences, Faculty of Human Sciences, Mid Sweden University, Sundsvall, Sweden

**Keywords:** BMI, overweight, shuttle run test, VO2max, waist circumference

## Abstract

This study aimed to examine: (i) the level of physical activity (PA), obesity indices and cardiorespiratory fitness (CRF) among boys and girls in primary school, and (ii) to determine the association of obesity indices and PA with CRF for the total number of participants, and then separately for boys and girls. 753 sixth to ninth grade girls and boys aged 10–14 years took part in this cross-sectional study. The PA was assessed by the “Physical Activity Questionnaire – Children” and CRF was assessed by the Maximal multistage a 20 m shuttle run test. Body mass index (BMI), waist circumferences (WC), and waist to height ratio (WHtR) were considered as obesity indices. Multiple linear regression analyses were performed to explore correlates of CRF. The results obtained showed the prevalence of general overweight and obesity was 25.5% in our sample which was lower than that in the regional estimate (e.g., ∼28%) for Eastern Europe. Among all participants, CRF was associated with male sex, older age, a lower WC percentile, higher WHtR, and higher level of PA. The model accounted for 24% of the variance. CRF was associated with older age and higher level of PA among girls and boys. Lower WC percentile was a significant determinant of CRF among boys. In conclusion, general overweight/obesity was not independently associated with CRF. Those with better CRF were more likely to be male and older, had a higher level of PA and lower central adiposity. These findings emphasize the importance of supporting school age children to take a part in programmed physical activity regardless of their body composition.

## Introduction

Child and adolescent obesity is a significant health problem worldwide (Flodmark et al., 2004; Gomes et al., 2014) reaching epidemic proportions both in developed (Ogden et al., 2002; Gomes et al., 2014) and less developed countries (Ebbeling et al., 2002). The prevalence of general overweight and obesity has been reported to be between 20 and 45% in European children (Lobstein and Frelut, 2003; Gomes et al., 2014, 2015) with a regional estimate of 28% for the eastern region of the European Union (Jackson-Leach and Lobstein, 2006). In the most recent report of the World Health Organization (2017), was shown that the prevalence of overweight and obesity continued to increase across most of European countries and regions in school children and adolescents (e.g., 11–15 years old) from 2002 to 2014. Furthermore, the report revealed differences in the prevalence between sexes (i.e., the average prevalence of 24% for boys and 14% for girls), between age groups (i.e., higher in younger age), and between the countries (i.e., the highest prevalence in southern European and Mediterranean countries) in 2014. However, the WHO’s data was based on self-reported height and weight which could underestimate the truth data (Elgar and Stewart, 2008). Comparing to the regional estimate the situation in Bosnia and Herzegovina is somewhat better with the reported prevalence of ∼21%, but it should be noted that the study was conducted with schoolchildren from only one canton in the country (Hasanbegovic et al., 2010). Unfortunately, the major concern with children obesity is that 80% of obese children become obese adults (Whitaker et al., 1997) and that overweight children and adolescents show an increased rate of mortality due to cardiovascular and digestive diseases in adulthood (Mossberg, 1989). Additionally, obese children are at increased risk to be diagnosed asthma at school age (Loid et al., 2015), they are more likely to have extremity fractures, to die of traumatic injuries than nonobese children (Kim et al., 2016) and to have a lower cardiorespiratory fitness (CRF) in childhood (Tuan et al., 2018) and more than 10 years later (Pahkala et al., 2013).

In that regard, there are several indices which are frequently used to assess obesity in children, including body mass index (BMI), waist circumference (WC), and waist to height ratio (WHtR). Although BMI has been commonly used as a sensible indicator of overall adiposity in children (Ogden et al., 2002), it seems that BMI has limitations in assessing the prevalence of age- and sex-specified obesity as well as body composition and fat distribution (Savva et al., 2000). On the contrary, WC and WHtR have been reported to be stronger indicators of central obesity and better predictors of cardiovascular disease risk factors (e.g., high levels of plasma lipids, lipoprotein levels) in children (Savva et al., 2000). In this regard, all these indicators of obesity are recommended to be used in epidemiological studies as they are low-cost and simple to measure.

Furthermore, it has been suggested that a low level of physical activity (PA) is one of the main cause of children’s obesity (Page et al., 2005) and worldwide the forth risk factor related with mortality (World Health Organization, 2009). However, obesity may cause physical inactivity and not to be a consequence (Metcalf et al., 2011). Physically active children compared to inactive children are less susceptible to the risk factors of chronic diseases and obesity, and they are also more likely to stay physically active with a higher level of CRF during adolescence and adulthood (Janz et al., 2000). Even though it was assumed that PA and CRF are, similarly, related to obesity indicators, some previous studies have reported inconsistent findings suggesting that CRF is more related to abdominal adiposity (measured by WC) compared to the level of PA (Ortega et al., 2007). Additionally, it is shown that the level of CRF in childhood and adolescence is a better predictor of cardiovascular diseases (CVD) in adulthood compared to the level of PA *per se* (Janz et al., 2002), so it is recommended that the both correlates should be permanently and independently monitored in children (Esmaeilzadeh et al., 2013). However, comparing to anthropometric indicators of obesity (BMI, WC, WHtR), some studies reported CRF to be weaker predictor of CVD in children (Goncalves et al., 2015), which emphasize the importance of concurrent assessment of all three predictors (i.e., anthropometrics, PA and CRF) of CVD whenever is possible.

Moreover, studies frequently report inconsistent results on the prevalence of obesity in boys and girls, as well as a gender specific association between obesity indicators, PA and CRF levels in children (Al-Nakeeb et al., 2007; Ostojic et al., 2011). In that regard continuous monitoring of these items is very important for researchers and health care providers in order to develop adequate and gender specific obesity prevention and intervention strategies (Esmaeilzadeh et al., 2013). To our knowledge, there is only one study that systematically investigated the prevalence of overweight and obesity, and PA level in school-age children (i.e., 6–15 years) from Bosnia and Herzegovina (Hasanbegovic et al., 2010). However, even though the study provides valuable data, it involved only children from one part of the country (i.e., Sarajevo canton) and did not investigate the predictors of CRF. Moreover, the aforementioned WHO’s report (World Health Organization, 2017) did not include data from Bosnia and Herzegovina. To fill the gap, current study aims were (i) to separately examine the level of PA, obesity indices and CRF among boys and girls in primary school, and (ii) to determine the association of obesity indices and PA with CRF for the total number of participants, and then separately for boys and girls. We hypothesized that children with a normal weight and higher level PA would have a higher level of CRF.

## Materials and Methods

### Study Design, Participants, and Procedure

This cross-sectional study recruited 753 sixth to ninth grade school children aged 10–14 years between February and May 2015. With an assumed prevalence of obesity and overweight of 25% and precision of 0.05, the calculation indicated that the required sample size was around 288 per group (Pourhoseingholi et al., 2013). Geographically stratified random sampling was used. Ninety-seven primary schools were identified from four main and biggest urban areas in the Federation of Bosnia and Herzegovina (i.e., Sarajevo, Tuzla, Zenica, and Mostar). Rural schools that belong to the named municipalities were not included in the selection process to avoid the potential socio-economic differences between the areas. Eight schools were randomly selected from each of the four areas for the study. From each selected school, one randomly selected class (sixth to ninth grade) was included in the study with the final number of schools (classes) being 32 (4 areas × 8 schools/classes). Eight classes per grade were included in the study to maintain the proportion of grades, age and gender. A total number of 1025 students from 32 classes were invited to participate in the current study. The randomization process of the identified schools and classes was generated by the Excel 2003. Socio-economic data of the selected urban areas and schools were not collected and considered in the current study.

Principals and physical education teachers of the schools were informed about the study aim and methodology and once they agreed for their schools to take part in the study, an informative letter and medical history questionnaire were distributed to parents or guardians and a short briefing session was organized for interested parents at the schools. Written informed consent was received from all children and their parents or guardians after a detailed verbal and written explanation of the purpose of the study, experimental design, testing protocols, research benefits and potential risks of the study were provided to them. Children were informed that they were free to withdraw from the study at any time without consequences. Only children who provided their own and parents’ or guardians’ written informed consent were included in the study. The study was approved by the Tuzla University Ethics Committee (02/11-2842/14-3) and conformed to the principles of the Declaration of Helsinki on human experimentation (World Medical Association, 2013).

Only healthy children aged 10–14 years old were included in the study. Those who reported that they used any medication or had a history of neuromuscular or heart disease or injuries, and or had any limitations in PA for the previous 6 months, were excluded from the study. Children who were regularly involved in some kind of sports activity were asked to refrain from this training and to avoid sleep deprivation for at least 2 days prior to the testing sessions. The participants were asked to consume a light meal at least 3 h prior to the beginning of testing (i.e., VO2max testing) and to make sure that they were properly hydrated before and during testing.

### Data Collection

All data collection for one child was conducted in 1 day in three separate stages between 8 and 12 am. First, the level of PA was evaluated by a self-reported questionnaire and took place in the schools’ classrooms. The questionnaire sheet was given to each child with clear instructions on how to complete it by an experienced research staff member. The children were instructed to independently fill in the questionnaire within 30 min time framework. The same researcher supervised the data collection in the classroom. Afterward, all anthropometric measurements were taken in the changing rooms of the gym. In the final phase, the maximal oxygen uptake was estimated for each child in the schools’ gyms. The measurements were conducted in the gender-specific groups of 12–15 children.

#### The Level of Physical Activity

To evaluate the level of PA during the childrent’s leisure time, the Physical Activity Questionnaire–Children (PAQ-C) was used (Crocker et al., 1997; Kowalski et al., 1997). The self-reported questionnaire was developed to assess levels of moderate to vigorous PA in children from grade 4 upward. It showed to be reliable and valid tool in assessing level of activity in children (Crocker et al., 1997; Janz et al., 2008). The questionnaire consists of nine questions (items) specifically evaluated on a 5-point Likert type scale, with higher scores indicating a higher level of PA. The mean of the nine items was used in the calculation of the summary of total activity which is the composite score that can range from 1 to 5 (Kowalski et al., 1997). To overcome the language barrier, we used the Croatian translated version of PAQ-C that was understandable for the selected participants in Bosnia and Herzegovina speaking area too. The version showed satisfactory internal consistency value (0.80) in assessing the level of PA in children (Samarzija and Misigoj-Durakovic, 2013).

#### Anthropometric Data

To estimate the children’s anthropometric characteristics and obesity indices, the subjects were asked to remove their shoes and socks and to be with underwear only. All measurements were taken by the same trained operator following standard procedures. The following anthropometric variables were measured: body height (BH), body weight (BW), waist circumference (WC). Based on these measures, we calculated the BMI for each child [body weight (kg)/body height squared (m^2^)], BMI percentile, and WHtR.

Body height was measured to the nearest 0.01 m with a portable stadiometer (Astra scale 27310, Gima, Italy). Body mass was measured using a bioelectric body composition analyzer (Tanita TBF-300 increments 0.1%; Tanita, Tokyo, Japan). To define underweight, overweight and obese according to BMI, the 5th, 85th and 95th BMI for age, the Centers for Disease Control and Prevention (CDC) reference percentiles were used (Ogden et al., 2002; Goncalves et al., 2015). Waist-circumference (WC) was measured using a flexible measuring tape at the midpoint between the superior edge of the iliac crest and the inferior border of the ribcage, with the average of three measurements used in analysis (Goncalves et al., 2015). WC and WHtR were used as indirect measures of the amount of abdominal fat. For the WC, values above 85th percentile (Taylor et al., 2000) and a cut off of 0.5 for WHtR (McCarthy and Ashwell, 2006) have been used to identify central obesity in children.

#### Cardiorespiratory Fitness

Maximal aerobic power (VO2max) as a CRF level indicator was estimated by the Maximal Multistage 20 Meter Shuttle Run Test (Leger and Lambert, 1982). The test consisted of a shuttle running at a pre-set pace by the shuttle run test protocol and played on a CD recorder. In the test, the participant ran 20 m long shuttles after a signal was sounded. At the start of the test, the participant had to run at a speed of 8 km/h to reach the opposite line before another signal was given. The running speed increased every minute by 0.5 km/h. When the participants were unable to maintain the pace, that is when they failed to reach the lines with the audio signals on 2 consecutive occasions, or when they stopped because of fatigue, the last shuttle covered was used to estimate the maximal oxygen uptake (VO2max). The VO2max was estimated from the following equation: VO2max = 3.46 ^∗^ (L + NS/(L ^∗^ 0.4325 + 7.0048)) + 12.2, where L is the reached level and NS is a number of shuttles covered at the respective level. The test has shown to be a valid and reliable tool for the evaluation of maximal aerobic power in children (Van Mechelen et al., 1986). The participants were asked to perform 7 min running based warm-up at low intensity. They started with the test 2 min after the warm-up. The test was performed in groups of 12–15 children. Verbal encouragement was provided for all participants during the test.

### Statistical Analyses

Categorical variables (e.g., gender) were presented as absolute frequencies and percentages, while continuous variables by mean and standard deviation. In bivariate analyses, the independent *t*-test and the Mann–Whitney *U*-test ware used to compare girls and boys in continuous and ordinal variables, respectively. Differences across the age groups and body weight categories (based on BMI percentiles) were assessed using one-way ANOVA for continuous variables (e.g., VO2max) and the Kruskal–Wallis test for ordinal variables (e.g., PA level). When statistically significant difference between the groups was detected, pairwise comparisons were performed using a Bonferroni *post hoc* test to investigate where the difference lies. We performed multiple linear regression analyses to determine whether there was any association between independent variables (i.e., obesity indices and PA) and CRF (dependent variable) once for the total sample, and then separately for girls and boys controlling for other potential confounders (e.g., age). Altogether three multiple regression models were calculated. The data was presented in the form of coefficient (β), 95% Confidence Intervals, and *p*-values. The significance level for all statistical tests was set at *p* ≤ 0.05. All statistical analyses were completed with Microsoft Excel (2003) and PASW statistic package 24.0 (IBM/SPSS Inc., Chicago, IL, United States).

## Results

After the selection process, a total of 1025 students from 32 classes aged 10–14 years were invited to participate in this study, of whom 753 (361 girls and 392 boys) consented and were enrolled which resulted in a 73.5% participation rate. About 48% of participants were girls.

### Girls vs. Boys: Obesity Indices, Physical Activity, and Cardiorespiratory Fitness

The prevalence of general overweight and obesity (based on BMI) was 25.5% while the prevalence of central obesity (based on WC) was 16% among the total sample. As shown in Table 1, about 16% of girls and 18% of boys were overweight. Whereas, obesity was observed among 6.4% of girls and 10.9% of boys (*p* = 0.038). The prevalence of central obesity (waist circumference percentile ≥85%) was 19% and 13% among boys and girls, respectively (*p* = 0.029). Compared to boys, girls were shorter (*p* < 0.001) with a smaller waist circumference (*p* = 0.016), and had a lower level of PA (*p* < 0.001). Moreover, girls scored lower than boys in CRF (*p* < 0.001). Otherwise, there were no differences in weight, BMI percentile, and weight to height ratio between girls and boys.

**Table 1:** Students’ *t*-test and chi-square tests comparing age, anthropometric data, obesity indices, physical activity, and fitness among primary school age girls and boys (a total sample).

Variables	Girls		Boys		*p*
			
	Mean (*SD*)	n (%)	Mean (*SD*)	n (%)	
Total		361 (47.9)		392 (52.1)	
Age (years)	12.47 (1.06)		12.38 (1.09)		0.236
Height (meters)	1.60 (0.08)		1.63 (0.11)		<0.001
Weight (Kg)	53.27 (11.68)		54.96 (13.90)		0.074
BMI-percentile	59.75 (27.45)		58.22 (30.88)		0.473
BMI categories^a^					0.075
Under-weight		12 (3.3)		14 (3.6)	
Normal		268 (74.2)		265 (67.6)	
Over weight		58 (16.1)		70 (17.8)	
Obese		23 (6.4)		43 (10.9)	
Waist circumference (cm)	73.35 (8.90)		75.06 (10.33)		0.016
Central obesity^b^		47 (13.0)		74 (18.9)	0.029
Waist to height ratio	0.46 (0.05)		0.47 (0.28)		0.278
Level of PA	2.91 (0.63)		3.16 (0.65)		<0.001
Normal^c^ – level of PA	3.33 (0.43)	207 (57.3)	3.48 (0.44)	280 (71.4)	
Low^d^ – level of PA	2.34 (0.34)	154 (42.7)	2.37 (0.34)	112 (28.6)	
Cardiorespiratory fitness^e^	32.08 (4.85)		35.86 (6.46)		<0.001
Normal^f^ – cardiorespiratory fitness	39.75 (3.12)	62 (17.2)	46.3 (2.91)	67 (17.1)	
Low^g^ – cardiorespiratory fitness	30.49 (3.41)	299 (82.8)	33.71 (4.63)	325 (82.9)	


*SD, standard deviation; n, number; BMI, body mass index. ^*a*^Underweight, overweight and obese according to the 5th, 85th and 95th BMI percentiles. ^*b*^Waist circumference percentile ≥ 85%; PA, physical activity (1–5 point scale). ^*c*^PA ≥ 2.73. ^*d*^PA ≤ 2.73. ^*e*^based on VO_2_ max (ml ⋅ kg^-1^ ⋅ min^-*1*^). ^f^VO2max ≥ 37.0 (for girls) and VO2max ≥ 42.1 (for boys). ^*g*^VO2max ≤ 37.0 (for girls) and VO2max ≤ 42.1 (for boys).*

**FIGURE 1:**
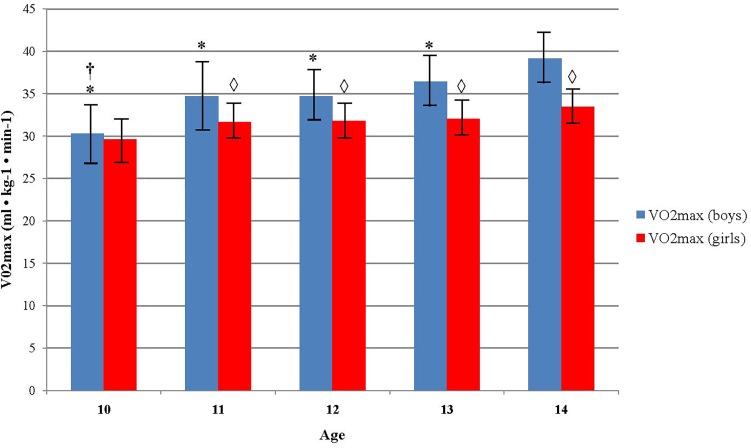
Differences in aerobic fitness level across age and sex. ^∗^Indicates values significantly different from those obtained in 14 years old boys at *p* < 0.05; ^†^Indicates values significantly different from those obtained in 13 years old boys at *p* < 0.05; ^
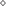
^Indicates significant differences between boys and girls in accompanied age group at *p* < 0.05.

There was a significant increase in VO2max across age groups for boys *F*(4, 387) = 9.1, *p* < 0.005, η^2^ = 0.086. VO2max increased from age 10 (30.27 ± 3.83), to age 11 (34.66 ± 6.48), to age 12 (34.67 ± 4.79), to age 13 (36.45 ± 7.15), and to age 14 (39.15 ± 6.20). The same pattern was observed among girls, but without significant differences observed between the age groups (*p* = 0.063). Boys showed to have significantly higher CRF comparing to girls in age groups 11–14. The mean differences between them increased with the age. At age 11 it was 3.08 ml ⋅ kg^-1^ ⋅ min^-1^ (95% CI, 1.19–4.96), *t*(144) = 3.23, *p* = 0.002, at age 12 it was 2.91 ml ⋅ kg^-1^ ⋅ min^-1^ (95% CI, 1.60–4.21), *t*(209) = 4.04, *p* = 0.00, at age 13 it was 4.41 ml ⋅ kg^-1^ ⋅ min^-1^ (95% CI, 2.83–6.00), *t*(238) = 5.49, *p* = 0.00, and at age 14 it was 5.85 ml ⋅ kg^-1^ ⋅ min^-1^ (95% CI, 3.77–7.63), *t*(132) = 5.85, *p* = 0.00 (Figure 1).

**FIGURE 2:**
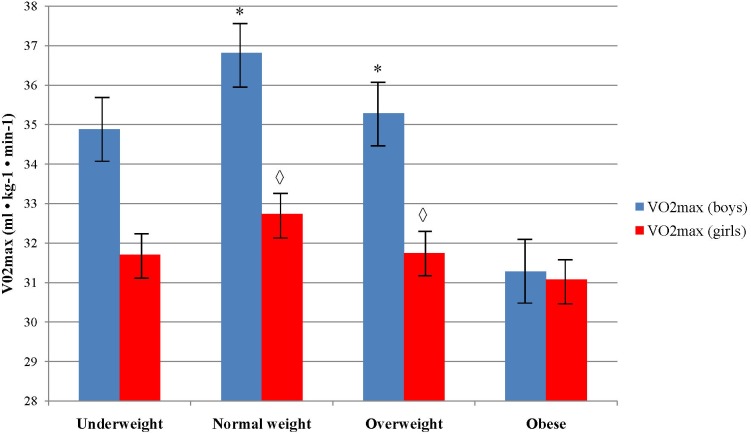
Differences in aerobic fitness level across body weight categories and sex. ^∗^Indicates values significantly different from those obtained in obese weight category among boys at *p* < 0.05; ^
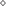
^Indicates significant differences between boys and girls in accompanied body weight category at *p* < 0.05.

Boys who were classified in normal and overweight body weight group showed to have a higher VO2max (36.81 ± 6.56 and 35.28 ± 5.31, respectively) comparing to those who were in underweight and obese group (34.88 ± 7.13 and 31.27 ± 5.01, respectively), *F*(3, 388) = 9.9, *p* < 0.001, η^2^ = 0.071. There were not significant differences in VO2max between the body weight categories in girls (*p > 0.05*). Boys from normal and overweight category had higher VO2max (36.81 ± 6.56 and 35.28 ± 5.31, respectively) comparing to girls (32.13 ± 4.86 and 31.74 ± 4.09, respectively), while there were not significant differences between sexes in obese and underweight group (Figure 2).

**FIGURE 3:**
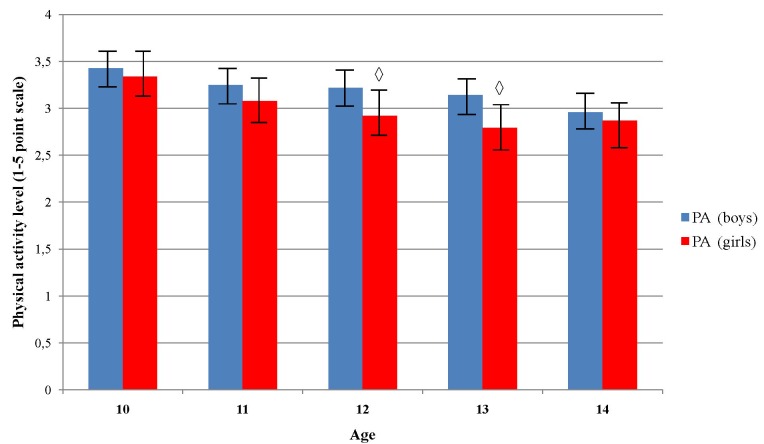
Differences in physical activity level across age and sex. ^
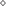
^Indicates significant differences between boys and girls in accompanied age group at *p* < 0.05.

**FIGURE 4:**
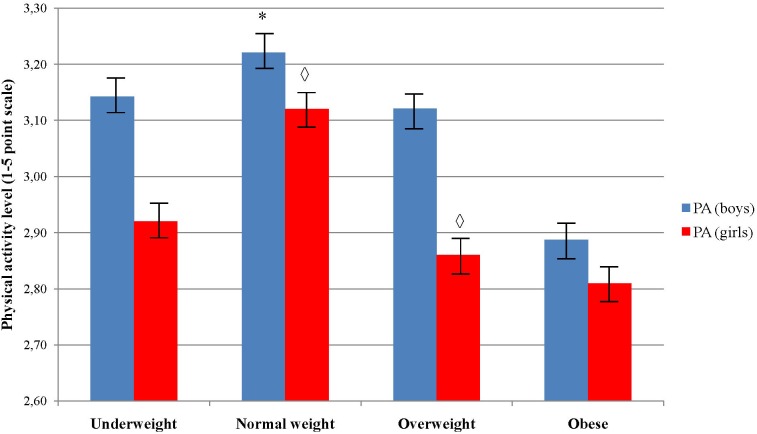
Differences in physical activity level across body weight categories and sex. ^∗^Indicates values significantly different from those obtained in obese weight category among boys at *p* < 0.05; ^
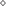
^Indicates significant differences between boys and girls in accompanied body weight category at *p* < 0.05.

PA level significantly decreased across age groups in girls (*p* = 0.010), but not in boys (*p* = 0.086) (Figure 3). *Post hoc* test did not reveal any significant pairwise differences among age groups in girls (Figure 3). Boys showed to be significantly more physically active than girls in age 12 and 13 (*p* = 0.00 and *p* = 0.01, respectively). Boys who were classified in normal body weight group showed to have higher level of PA comparing to those who were in underweight, overweight and obese group, χ^2^(3) = 9.29, *p* = 0.026 (Figure 4). Girls did not showed significant differences in PA level across the body weight categories, χ^2^(3) = 1.97, *p* = 0.579. Boys from normal and overweight category were physically more active comparing to girls (*p* < 0.01), while there were no significant differences between them in obese and underweight group (*p* > 0.05) (Figure 4).

### Factors Associated With Cardiorespiratory Fitness

Visual inspection of a normal probability plot showed that residuals were normally distributed. Analyses among all participants (*n* = 753) revealed that better CRF was associated with male sex (*p* < 0.001), older age (*p* < 0.001), lower waist circumference percentile (*p* = 0.034), higher weight to height ratio (*p* = 0.045) and higher level of PA (*p* < 0.001). The model accounted for 24% of the variance of CRF.

As shown in Table 2, CRF was associated with older age and a higher level of PA among primary school age girls and boys. For boys, having lower waist circumference percentile was also a significant determinant (*p* = 0.048). The model could explain 22% of the variance of CRF among boys and 10% among girls, respectively.

**Table 2:** Factors associated with cardiorespiratory fitness among all participants (*n* = 753) and separately for girls (*n* = 361) and boys (*n* = 392) in multiple linear regression analyses.

Independent variables	Total sample		Girls		Boys	
			
	β	95% CI	β	95% CI	B	95% CI
Sex						
Boys						
Girls	-0.28	-4.12 to -2.54^∗∗∗^				
Age	0.30	1.23–2.08^∗∗∗^	0.22	0.49–1.54^∗∗∗^	0.39	1.64–2.94^∗∗∗^
BMI percentile	-0.07	-0.03 to 0.01	0.01	-0.02 to 0.03	-0.10	-0.05 to 0.01
Waist circumference percentile	-0.16	-0.06 to -0.002^∗^	-0.15	-0.07 to 0.01	-0.20	-0.09 to 0.00^∗^
Waist to height ratio	0.14	0.001–0.06^∗^	0.16	-0.01 to 0.06	0.16	-0.00 to 0.08
Physical activity level	0.30	2.21–3.42^∗∗∗^	0.29	1.44–2.98^∗∗∗^	0.32	2.32–4.13^∗∗∗^
Total *R*^2^	0.243		0.105		0.220	


*CI, confidence interval; BMI, body mass index; β, a standardized beta coefficient; R^*2*^, the coefficient of determination; ^∗^p < 0.05; ^∗∗^p < 0.01; ^∗∗∗^p < 0.001.*

## Discussion

The aim of the present study was twofold. Firstly, it attempted to separately examine the level of PA, obesity indices and CRF among boys and girls in primary school, and secondly to determine the association of obesity indices and PA with CRF for all participants and then separately for boys and girls. The results revealed several important findings that should be emphasized here. First, even though the results showed relatively low prevalence of overweight/obesity and relative a high number of children who met recommended PA time, it is a worrying fact that more than 80% of them had a low CRF which put them at high metabolic risk. Secondly, although boys were more frequently obese than girls, girls had a lower level of PA and CRF. Thirdly, sex, age, PA level and central adiposity showed to be independent determinants of CRF among school age children.

Before discussing these findings, we will provide a brief overview of the established prevalence. The prevalence of general overweight and obesity was 25.5% in our sample which was lower than that in the regional estimate (e.g., ∼28%) for Eastern (Jackson-Leach and Lobstein, 2006) and 30.3–45% Southern Europe (Gomes et al., 2014), but higher than it was reported for schoolchildren from Sarajevo Canton in Bosnia and Herzegovina (21%) (Hasanbegovic et al., 2010). However, Ostojic et al. have reported an even higher prevalence of obesity (e.g., ∼39%) for Serbian school age children from 6 to 14 years (Ostojic et al., 2011). The central obesity, which was shown to be correlated with CVD risk factors and unfavorable metabolic profiles in children and adults (Savva et al., 2000), was observed among 16% of the current sample. Our results seem to suggest that even though overweight and obesity among Bosnian children, is somewhat lower than in other developing/developed countries (Jackson-Leach and Lobstein, 2006; Ostojic et al., 2011; Gomes et al., 2014, 2015), it is still high and should be considered as a potential public health concern. This should be taken into account by policy makers in Bosnia and Herzegovina investing in health education of children, to help these children maintain an appropriate weight to decrease adverse health effects in adulthood.

In the current study, girls had lower values in obesity indices than boys, which was in accordance with some previous studies (Ortega et al., 2007; Gomes et al., 2014, 2015; World Health Organization, 2017; Tuan et al., 2018) and at odds with others (Al-Nakeeb et al., 2007; Ostojic et al., 2011; Dencker et al., 2012). Lower obesity indices among girls in this age range may not necessarily pertain to their healthier lifestyle as our results showed that girls had a lower level of PA and CRF compared to boys across all age groups with a more prominent difference in age of 12 and 13. Likewise, Gomes et al. (2017) have reported that boys participate in more PA than girls with a trend of decreasing the level of PA being more rapid in girls compared to boys in the 11–13 year age group (Armstrong et al., 2000). Furthermore, one could argue that our findings could be related to girls’ nutritional status, as the girls at this age might be more concerned about their body image (Mond et al., 2011). This is an issue which also needs to be considered in studies assessing health behavior among school age children. However, the differences in age range, ethnicity and socio-cultural factors may likely explain the inconsistent results in the above mentioned studies.

Our findings were in agreement with research revealing that school age boys performed better in the maximal multistage fitness test across all age groups and therefore they had a higher estimated VO2max (Ruiz et al., 2007; Ostojic et al., 2011; Dencker et al., 2012; Tuan et al., 2018). PA level, body composition (greater muscle mass), cardiac size and function as well as mechanical efficiency (e.g., larger levers) may explain the gender differences in cardiovascular fitness among children (Rowland et al., 2000; Manna, 2014). Moreover, in the current sample, based on suggested cut-off values (2.73) for PAQ-C (Benítez-Porres et al., 2016), a higher percentage of girls 43.2% (*N* = 156) did not meet recommended MVPA (moderate-vigorous PA) time (>60 min) (Strong et al., 2005) comparing to the boys, 28.8% (*N* = 113), which is in line with some recently published studies (Gomes et al., 2015, 2017; World Health Organization, 2017). In this context, the discrepancy in MVPA between the sexes could additionally explain their differences in CRF too.

Furthermore, it is reported (e.g., Ostojic et al., 2011; Esmaeilzadeh et al., 2013; Tuan et al., 2018) that there is a negative correlation between central obesity (WC and WHtR) and cardiovascular fitness which is in line with our findings. Those children with higher central obesity have shown to have lower CRF estimated by using the maximal multistage 20 m shuttle run test. The negative relationship can be explained by the test’s “stop and go” nature that continuously required subjects to accelerate and decelerate while overcoming their body inertia which is more demanding in obese subjects who have more fat as a nonfunctional, ballast mass (Katic, 1996). On the other side, there was not a difference in VO2max between boys who belong to the normal and overweight group (estimated by BMI) which possibly indicates a higher level of fat free mass in the overweight group and point out the limitation of BMI to differentiate fat and muscle mass in children (Vanderwall et al., 2017). This was a pattern which was observed only among boys in our study. The multivariate analyses of our sample showed that none of the obesity indices were independently associated with CRF among girls. On the contrary, the higher level of PA was positively associated with better CRF in the both groups which is consisted with other contemporary data on school age children (Esmaeilzadeh et al., 2013; Chen et al., 2018). Namely, Chen et al. (2018) showed that fifth-grade students, both girls and boys, who had bigger total PA time and were more engaged in organized PA had better developed CRF and muscular endurance. In particular, our findings emphasize the importance of encouraging school age children to do PA independent of their level of obesity or weight, as PA directly improves their physical fitness that is associated to many health-related benefits at all ages (Ortega et al., 2008; Chen et al., 2018). Moreover, comparing to WC, BMI has not been correlated to CRF which emphasize the importance of regular measurements of central obesity. This finding confirms the importance of WC measurement that has been proved to be a stronger predictor of cardiovascular disease risk factors (Savva et al., 2000) than BMI and emphasize beneficial effects of CRF on children health.

Furthermore, the recent studies showed a negative relationship between PA, MVPA and obesity indices (e.g., BMI, WC, body fat percentage) (Gomes et al., 2015, 2017). Unfortunately, it was reported that only few children achieve the sustained period of moderate to vigorous PA (Al-Nakeeb et al., 2007; World Health Organization, 2017), recommended by guidelines to maintain good health (World Health Organization, 2006; Tremblay et al., 2011), and the situation is even worse among overweight and obese children (Dorsey et al., 2011). In the current sample, even though 64.3% of children met the recommended PA time, only 17.1% (*N* = 67) boys and 17.2% (*N* = 62) girls were at a hypothetically low metabolic risk according to established cut-off values for VO2max level of 37.0 and 42.1 ml/kg/min in girls and boys, respectively (Ortega et al., 2008). In this regard, only advising or educating the children to do PA is not equivalent to doing an effective activity leading to better physical fitness. Thus, obesity preventive interventions should focus on designing physical activities that would increase MVPA time (e.g., high-intensity interval based activities) which has been proved to improve children’s physical fitness effectively (Racil et al., 2016) and reduce obesity indices such as BMI scores (Bingham et al., 2013; Trinh et al., 2013; Burke et al., 2014). Another key point to remember is that obesity may cause physical inactivity and not to be a consequence (Metcalf et al., 2011). This can lead to a vicious circle. That is to say, physical inactivity may negatively affect skills acquisition and execution required for many sport and physical activities which, as a result, has less self-confident children who are discouraged from being a part of such activities and consequently are at higher risk to gain a weight and lower fitness level (Pietiläinen et al., 2008; Esmaeilzadeh et al., 2013; Pahkala et al., 2013).

We have found that the older age was associated with better cardiovascular fitness, which is in line with older (Houlsby, 1986) and recent studies (Tuan et al., 2018) that showed that maturation positively affected CRF in children. The results could pertain to the growth, increase in muscle mass, strength and mechanical efficiency, and maturation of the cardiovascular system (Rogol et al., 2000; Manna, 2014). Or simply, we can speculate that those who were older could perform multistage fitness test more precisely. Contrary, one may suggest that by aging, children may have a better self perception and perception of health and health behavior benefits (Strong et al., 2005) which in turn can lead to the higher level of PA and CRF, respectively. However, we found the level of PA decreased progressively with age for both boys and girls, but more rapidly in girls at age 12 and 13 years, which was in line with a study by Telama and Young showing a remarkable decline in frequency of PA after the age of 12 (Telama and Yang, 2000).

### Limitations

This study has provided new insights into the physical health of school age children in Bosnia and Herzegovina. The strengths of the study were a relatively large sample size which was selected randomly from four urban areas in the Federation of Bosnia and Herzegovina, implying that this sample may represent the entire urban population of students at age 11–14 years. However, the study has several limitations which must be acknowledged. The cross-sectional characteristic of the data does not allow the establishment of firm causal links. The optimal study design to predict the CRF from PA and obesity indices would be a longitudinal follow-up research design. Moreover, the level of PA was assessed by a subjective measure. Even though the questionnaire PAQ-C is widely used and has a good reliability and validity (Kowalski et al., 1997; Janz et al., 2008; Samarzija and Misigoj-Durakovic, 2013), no objective measures were taken to verify students’ responses. Furthermore, PAQ-C was originally developed to assess general levels of PA and does not provide information regarding the caloric expenditure, frequency, time, and intensity of the activity. Although, the age of the selected sample was likely to differ in the maturation, we did not assess the pubertal stage which somewhat hindered us in clarifying and interpreting our data regarding sex differences in obesity, PA and CRF that we have observed. Moreover, we did not directly measure body fat percentage, but we used BMI to categorize the children in different body weight groups (e.g., overweight and obese) which could lead to unreliable data in terms of the accurate estimation of children’s body composition and the group affiliation. Additionally, these results may not be generalizable to school age children living in rural areas too. Moreover, the amount of explained variance in the models was small and yet more not so important because a lot of variance in the model might be explained by the confounding variables that were not controlled. It indicates that further investigations should include other possible confounding variables (e.g., socio-cultural and ethno-religious characteristics, nutritional behavior, screen time, sedentary time, maturation etc.), which in return could explain the roots of the obesity indices and PA level in the sample and thus reveal their cause-and-effect relationship with the CRF.

## Conclusion

In conclusion, even though the results showed relatively low prevalence of overweight/obesity (comparing to regional estimate) and relative a high number of children who met recommended PA time in the current study, it is a worrying fact that more than 80% had a low CRF that put them at high metabolic risk. Moreover, this study demonstrated that general overweight/obesity was not independently associated with CRF. We observed that those with better CRF were more likely to be male, to be older and to have a higher level of PA. Therefore, it is important to support school age children, especially girls, to take a part in programmed PA regardless of their body composition.

Furthermore, elementary schools appear to be an ideal environment to implement a holistic obesity preventive strategies with physical education (PE) being the best tool in keeping children physically active during a day (Burke et al., 2014). In light of our study, this is even more important, when we know that children who participated in the present study had PE only two times a week for 45 min due to the school curriculum in Bosnia and Herzegovina, which is definitely not sufficient for inducing a health related benefits and combat against obesity. This should be taken into account by policy makers investing in physical and health education of children, to help these children maintain an appropriate weight to decrease adverse health effects in adulthood. Preventive strategies should target two main causes of children obesity: poor diet (increased energy intake) and a lower level of PA (decreased energy expenditure) (Anderson and Butcher, 2006).

## Data Availability

The datasets for this study can be found at: https://www.dropbox.com/s/2grbsuoawn005hd/Data-primary-school%20-%20DROPBOX.xls?dl=0.

## Author Contributions

HP conceptualized and designed the study and organized and supervised data collection. BE undertook the data analysis and interpretation. HP and BE led together the writing of the paper.

## Conflict of Interest Statement

The authors declare that the research was conducted in the absence of any commercial or financial relationships that could be construed as a potential conflict of interest.
